# The Impact of Paratracheal Lymphadenectomy on Survival After Esophagectomy: A Nationwide Propensity Score Matched Analysis

**DOI:** 10.3390/cancers17050888

**Published:** 2025-03-05

**Authors:** Eliza R. C. Hagens, B. Feike Kingma, Mark I. van Berge Henegouwen, Alicia S. Borggreve, Jelle P. Ruurda, Richard van Hillegersberg, Suzanne S. Gisbertz

**Affiliations:** 1 Department of Surgery, Cancer Center Amsterdam, Amsterdam University Medical Centers, University of Amsterdam, De Boelelaan 1117, 1081 HV Amsterdam, The Netherlands; elizahagens@gmail.com (E.R.C.H.); m.i.vanbergehenegouwen@amsterdamumc.nl (M.I.v.B.H.); 2 Department of Surgery, University Medical Center Utrecht, Heidelberglaan 100, 3584 CX Utrecht, The Netherlands; b.f.kingma-3@umcutrecht.nl (B.F.K.); a.s.borggreve-2@umcutrecht.nl (A.S.B.); j.p.ruurda@umcutrecht.nl (J.P.R.); r.vanhillegersberg@umcutrecht.nl (R.v.H.)

**Keywords:** esophageal cancer, paratracheal lymphadenectomy, esophagectomy

## Abstract

**Purpose:** To investigate the impact of paratracheal lymphadenectomy on survival in patients undergoing an esophagectomy for cancer. The secondary objective was to assess the effect on short-term outcomes. **Methods:** Between 2011–2017, patients with an esophageal or gastroesophageal junction carcinoma treated with elective transthoracic esophagectomy with two-field lymphadenectomy were included from the Dutch Upper Gastro-intestinal Cancer Audit registry. After 1:1 propensity score matching of patients with and without paratracheal lymphadenectomy within histologic subgroups, short-term outcomes and overall survival were compared between the two groups. **Results:** A total of 1154 patients with adenocarcinoma and 294 patients with squamous cell carcinoma were matched. Lymph node yield was significantly higher (22 versus 19 nodes, *p* < 0.001) in patients with paratracheal lymphadenectomy for both tumor types. Paratracheal lymphadenectomy was associated with more recurrent laryngeal nerve injury (10% versus 5%, *p* = 0.002) and chylothorax in patients with adenocarcinoma (10% versus 5%, *p* = 0.010) and with more anastomotic leakage in patients with squamous cell carcinoma (42% versus 27%, *p* = 0.014). The 3- and 5-year survival in patients with and without a paratracheal lymphadenectomy were for adenocarcinoma, respectively, 58% versus 56% and 48% in both groups (log rank: *p* = 0.578) and for patients with a squamous cell carcinoma, 62% in both groups and 57% versus 54% (log rank: *p* = 0.668). **Conclusions:** The addition of paratracheal lymphadenectomy significantly increases lymph node yield in transthoracic esophagectomy but did not result in improved survival for esophageal cancer patients in the current dataset. However, there was an increase in postoperative morbidity in patients who underwent a paratracheal lymphadenectomy.

## Simple Summary

This nationwide study investigated the impact of paratracheal lymphadenectomy on survival after esophagectomy. The study found that paratracheal lymphadenectomy increased the lymph node yield but also resulted in higher postoperative morbidity, including recurrent laryngeal nerve injury and chylothorax. Despite the increased lymph node yield, the hypothesized survival benefit of paratracheal lymphadenectomy was not seen. This study highlights the complexity of balancing surgical benefits and risks. While paratracheal lymphadenectomy increases lymph node yield, the associated postoperative complications outweigh potential survival benefits. Whether these results remain when paratracheal lymphadenectomy is performed as a standard procedure in all esophageal cancer patients requires further investigation.

## 1. Introduction

For patients with locally advanced esophageal cancer, neoadjuvant therapy followed by a radical esophageal resection likely offers the best chance of a cure [1,2]. Despite the addition of neoadjuvant therapy and the development of minimally invasive surgical techniques, the prognosis of esophageal cancer remains poor with a 5-year survival rate of 40–50% [3,4,5,6,7]. In addition to surgery, chemoradiation therapy is also a well-established treatment option, particularly for squamous cell carcinoma. There is ongoing debate regarding the optimal surgical approach for gastroesophageal junction tumors, especially for Siewert type 2 and 3 tumors, while for Siewert I cancers, a thoracic approach following an abdominal phase with perigastric D2-lymphadenectomy is widely accepted.^8^ The extent of lymph node involvement is a major predictor of long-term survival in esophageal cancer patients and recent studies demonstrated that increased total lymph node yield is associated with improved survival following esophagectomy [7,8]. However, studies on the impact of dissecting specific lymph node stations are scarce, especially for those located in the upper mediastinum [8,9,10,11].

Surgical resection by means of a transthoracic esophagectomy with a 2-field lymphadenectomy is the preferred intervention for esophageal cancer in the Netherlands and worldwide, while no consensus exists regarding the exact extent of the mediastinal lymph node dissection [6,7,8,9,10,11,12]. In particular, the routine dissection of the paratracheal lymph node stations has been a topic of debate. Although paratracheal lymphadenectomy might increase the likelihood of removing all nodal metastases, thereby achieving locoregional disease control and maybe improved survival, this potential benefit must be carefully weighed against the possible risks of increased morbidity by damage to structures such as the recurrent laryngeal nerves [9]. In this context, an Asian study suggested that paratracheal lymph node dissection has a significant positive impact on survival [13]. Furthermore, a recent nationwide study in the Netherlands demonstrated that paratracheal lymphadenectomy is associated with increased lymph node yield without increasing the incidence of recurrent laryngeal nerve injury or other complications [14]. Although these few studies seem to rule in favor of performing paratracheal lymphadenectomy in esophagectomy, more research is needed to further investigate and possibly quantify the hypothesized survival advantage. Therefore, the primary aim of this study is to investigate the impact of paratracheal lymphadenectomy on survival after esophagectomy for esophageal cancer. The secondary aim was to assess the effect of paratracheal lymphadenectomy on short-term outcomes.

## 2. Methods

### 2.1. Study Design

Data were retrieved from the Dutch Upper Gastro-intestinal Cancer Audit (DUCA) registry for this nationwide population-based cohort study. It is mandatory for hospitals to register all patients with esophageal and gastric cancer undergoing surgery with the intent of resection in the DUCA registry. The scientific committee of the DUCA approved this study and no informed consent or ethical approval was required under Dutch law.

### 2.2. DUCA Data and Survival Data Collection

In the DUCA registry, patient and treatment characteristics and postoperative outcomes are registered until 30 days after esophagectomy. As part of this registry, all Dutch hospitals are required to register whether or not a lymph node dissection was performed in the upper mediastinum (i.e., paratracheal, along the recurrent laryngeal nerves) and in the middle or lower mediastinum (i.e., subcarinal, paraesophageal, and around the pulmonary ligament). As the DUCA registry does not contain long-term follow-up data, the dataset was combined with a dataset provided by Vektis in September 2017. Vektis is a national health care insurance database including all medical treatments paid for by Dutch insurance companies [15]. This database registers the date of death in case any patient with health care insurance dies. Hence, patients who were not registered as being deceased at the time of merging the DUCA and Vektis databases (1 September 2017) are assumed to be alive. Since health care insurance is mandatory in the Netherlands, the Vektis database covers 99% of all Dutch inhabitants [15]. The cause of death is not recorded.

### 2.3. Patient Population

Patients entered in the DUCA registry were eligible for inclusion if they had a primary adenocarcinoma or squamous cell carcinoma of the esophageal or gastroesophageal junction (cT1-4aN0-3M0) and were treated by elective transthoracic esophagectomy with two-field lymphadenectomy between 2011 and 2017. Patients were excluded if they did not undergo at least a dissection of the subcarinal and paraesophageal lymph nodes during esophagectomy; if the extent of lymphadenectomy was missing; if they underwent a salvage esophagectomy; or if the resection was performed for recurrent disease.

### 2.4. Outcome Measures

Data regarding patient characteristics, treatment details, and postoperative outcomes were retrieved from the DUCA registry. The primary endpoint was the overall survival of patients who underwent paratracheal lymphadenectomy and patients who did not. Secondary outcome measures were also compared between these groups and included radicality (R0), (positive) lymph node yield, pathological nodal status (pN status), postoperative complications (overall and specifically recurrent laryngeal nerve injury, anastomotic leakage, pulmonary complications and chylothorax, as defined by the DUCA), re-interventions, and hospital stay.

### 2.5. Statistical Analyses

Propensity scores were used to match patients who received a paratracheal lymphadenectomy during esophagectomy with those who did not. Matching was performed in two subgroups: patients with adenocarcinoma and squamous cell carcinoma. Propensity scores were obtained from a logistic regression model and included variables that were expected to influence survival, since this was the primary outcome, namely, patient characteristics (i.e., age, gender, Body Mass Index, ASA score, comorbidities, tumor locationand cTNM stage) and treatment characteristics (neoadjuvant therapy, surgical approach, location of anastomosis, year of surgery). Next, one-to-one matched study groups were created using nearest-neighbor matching without replacement. The balance was assessed using the standardized mean difference (SMD). An SMD ≤ 0.1 was considered to indicate good balance [16]. Only variables with SMD ≤ 0.1 were finally used for propensity score matching, since matching on more variables would lead to a smaller study population.

After propensity score matching, Kaplan Meier curves were plotted and log rank tests were performed to compare the overall survival between patients with paratracheal lymphadenectomy and patients without paratracheal lymphadenectomy. The other endpoints were compared by means of chi-square tests (for categorical variables), independent samples *t*-tests (for normally distributed continuous variables), or Mann–Whitney U tests (for non-normally distributed continuous variables).

All statistical analyses were performed using SPSS version 25.0 (IBM Corp., Armonk, NY, USA) and R version 3.6.1 (http://www.R-project.org (visited 1 January 2019), “matchit” and “optmatch” packages). A *p*-value of <0.05 was considered statistically significant.

## 3. Results

### 3.1. Patient Population

**Figure 1:**
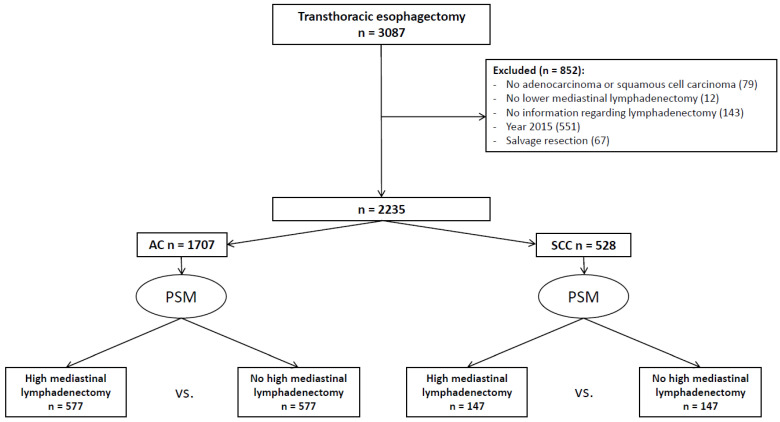
Inclusion flowchart. *Legend:* AC; Adenocarcinoma. SCC; Squamous cell carcinoma. PSM; Propensity Score Matching.

A total of 3087 patients undergoing an elective transthoracic esophagectomy between 2011 and 2017 were identified in the DUCA registry. A total of 79 patients were excluded because they had a tumor histology other than adenocarcinoma or squamous cell carcinoma, 12 patients were excluded because they did not undergo a lower mediastinal lymphadenectomy, 143 patients were excluded because the extent of the lymphadenectomy was missing, and 67 patients were excluded because they underwent a salvage resection. Finally, patients who underwent surgery in 2015 were excluded (*n* = 551), because the extent of lymphadenectomy was not registered in that year. The remaining 2235 patients were included for propensity score matching (Figure 1).

### 3.2. Adenocarcinoma

Before propensity score matching, 1707 patients had an adenocarcinoma of which 959 (56%) underwent a paratracheal lymphadenectomy. After propensity score matching, 1154 (577 patients in both groups) remained for further analyses. Patient and treatment characteristics before and after matching are shown in Table 1.

Table 2 shows the clinical and pathological results of patients with adenocarcinoma after esophagectomy. Patients with a paratracheal lymphadenectomy had a higher median number of total lymph nodes removed, 22 (IQR 17–30) compared to 19 (IQR 15–25, *p* < 0.001). The number of positive nodes, pN stage and R0-resection rate were similar in both groups. The incidence of overall postoperative complications was similar in both groups (61% in patients who underwent paratracheal lymphadenectomy versus 60% in patients who did not, *p* = 0.857). However, the incidence of recurrent laryngeal nerve injury was higher in the group of patients with paratracheal lymphadenectomy (10% versus 5%, *p* = 0.002). The incidence of chylothorax was also higher in patients with paratracheal lymphadenectomy (10% versus 5%, *p* = 0.010). The incidence of other complications, re-interventions, hospital stay, and postoperative mortality was comparable in both groups.

**Figure 2:**
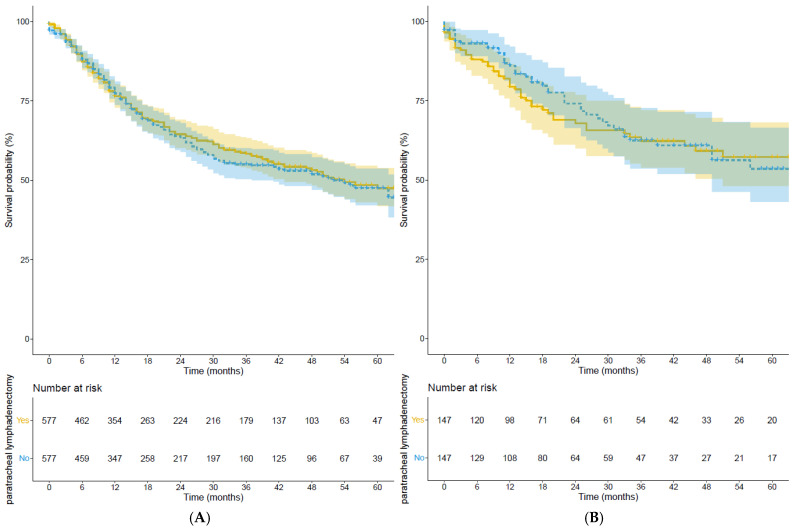
Overall survival for patients with adenocarcinoma (**A**) and squamous cell carcinoma (**B**) with and without high mediastinal lymphadenectomy.

Median overall survival for patients with paratracheal lymphadenectomy was 48 months (95%CI 45–51) compared to 46 months for patients without a paratracheal lymphadenectomy (95%CI 42–49), log rank: *p* = 0.578, Figure 2. The 3- and 5-year overall survival were, respectively, 58% and 48% for patients with paratracheal lymphadenectomy and 56% and 48% for patients without this dissection.

### 3.3. Squamous Cell Carcinoma

A total of 528 patients with squamous cell carcinoma were included, of whom 365 (69%) underwent a paratracheal lymphadenectomy. By means of propensity score matching, 294 patients (147 in each group) were matched and included in subsequent analyses. Patient and treatment characteristics before and after matching are shown in Table 3.

Short-term outcomes for patients with and without paratracheal lymphadenectomy are shown in Table 4. The median lymph node yield was higher in patients with paratracheal lymphadenectomy (22 versus 19, *p* = 0.010). The number of positive lymph nodes, pN stage and R0-resection rate did not significantly differ between both groups. Postoperative complications occurred in 66% of patients with a paratracheal lymphadenectomy compared to 61% in the patients without a paratracheal lymphadenectomy (*p* = 0.396). However, the incidence of anastomotic leakage was higher in patients with paratracheal lymphadenectomy compared to patients without paratracheal lymphadenectomy (42% versus 27%, *p* = 0.014). The incidence of recurrent laryngeal nerve injury, other complications, re-interventions, and postoperative mortality did not differ significantly between both groups. Length of hospital stay for patients with paratracheal lymphadenectomy was longer (14 days, IQR 16–30 versus 12 days, IQR 13–26, *p* = 0.004).

Median overall survival for patients with paratracheal lymphadenectomy was 52 months (95%CI 47–58) compared to 53 months in patients without paratracheal lymphadenectomy (95%CI 47–58). Log rank: *p* = 0.668, representing an insignificant difference, as shown in Figure 2. The 3- and 5-year overall survival were 62% and 57% for patients with paratracheal lymphadenectomy and 62% and 54% for patients without a paratracheal lymphadenectomy.

## 4. Discussion

This nationwide study assessed the impact of paratracheal lymphadenectomy on long-term survival for patients with esophageal adenocarcinoma and squamous cell carcinoma separately. Although paratracheal lymphadenectomy was associated with higher lymph node yield in both groups, no significant difference in survival was observed between patients who underwent paratracheal lymphadenectomy compared to patients who did not in patients with adenocarcinoma. Paratracheal lymphadenectomy was not associated with an increased overall morbidity rate. However, in patients with squamous cell carcinoma, paratracheal lymphadenectomy was associated with a higher anastomotic leakage rate and a longer length of hospital stay compared to patients without paratracheal lymphadenectomy. In patients with adenocarcinoma, higher incidences of recurrent laryngeal nerve injury and chylothorax were observed in patients who underwent a paratracheal lymphadenectomy.

The lymph node yield in our study seems lower than reported in other studies and may be attributable to a fewer number of centers that perform back-table dissection, paratracheal lymph node dissection, and no dissection of the thoracic duct compartment [17,18]. A higher lymph node yield has previously been reported to be associated with improved survival in esophageal cancer patients [17,19]. In this context, an Asian study compared the survival of patients who underwent limited versus total mediastinal lymphadenectomy, including paratracheal lymph node dissection [13]. This relatively small retrospective study included 129 patients with T1 squamous cell carcinoma and reported significantly better survival in the group who underwent total mediastinal lymphadenectomy [13]. Other Asian studies, mostly including squamous cell carcinoma patients, seem to support these findings and suggest that resection of the upper mediastinal lymph nodes has a significant therapeutic value concerning long-term survival [20,21]. Less evidence is available concerning the role of paratracheal lymphadenectomy in Western esophageal cancer patients, who are mostly diagnosed with adenocarcinoma. Two studies assessed the rate of nodal recurrence in the upper mediastinum among patients with gastroesophageal junction adenocarcinoma who underwent an esophagectomy without paratracheal lymphadenectomy, reporting upper mediastinal lymph node recurrence in 4–7% of patients [22,23,24]. Although this suggests a potential survival benefit of paratracheal lymphadenectomy in Western patients, this effect could not be objectified in the current study. It should be noted, however, that most Dutch centers only perform paratracheal lymphadenectomy in selected cases based on preoperative staging, if clinically suspected paratracheal lymph node metastases are observed. Therefore, patients who underwent paratracheal lymphadenectomy possibly had more extensive disease to begin with, with a worse prognosis. While paratracheal lymphadenectomy is a standard procedure in some centers, the majority of centers perform it only on indication. Although propensity score matching—including cTNM stage—was performed to minimize selection bias due to differences between the groups, we were unable to correct for all bias by indication. A possible survival benefit of paratracheal lymphadenectomy must be weighed against the risk of damaging the recurrent laryngeal nerves and thoracic duct if not ligated at the level of the diaphragm. Interestingly, this study only found a higher rate of recurrent laryngeal nerve injury and chylothorax in the group of patients with adenocarcinoma. Previous studies have been inconsistent regarding the impact of paratracheal lymphadenectomy on the incidence of recurrent laryngeal nerve injury, as some showed significantly increased rates while others did not find a difference [24,25,26]. In fact, a previous study with the Dutch nationwide database did not find an association between paratracheal lymphadenectomy and recurrent laryngeal nerve injury or chylothorax [14]. The contradictory findings in the current study, which was performed in a similar nationwide dataset, might be explained by a difference in methodology, as the previous study performed analyses in two separate groups based on surgical approach (Ivor Lewis and McKeown), while this study analyzed two groups based on tumor histology, as this is an important covariable in survival analysis for esophageal cancer (adenocarcinoma and squamous cell carcinoma). Additionally, among the patients who underwent paratracheal lymphadenectomy, the majority of those who experienced RLN injuries had a cervical anastomosis, which could have contributed to the higher incidence of this complication. Notably, even among patients who did not receive paratracheal lymphadenectomy, a significant number had a cervical anastomosis, which could similarly affect the observed rate of RLN injuries. Also, an additional and important factor to consider is the level of surgical expertise in performing paratracheal lymphadenectomy within the Dutch setting. In the Netherlands, this procedure is generally performed on an indication basis rather than as a routine component of esophagectomy. As a result, many Dutch surgeons may have limited exposure to and experience with paratracheal lymphadenectomy. This relative inexperience likely contributes to the observed higher incidence of RLN injuries [27,28,29]. This might indicate a need for more standardization. In the group of patients with squamous cell carcinoma, a higher incidence of anastomotic leakage was seen after paratracheal lymphadenectomy, which might be explained by a more extensive pleural dissection in the upper mediastinum. This might cause more intrathoracic manifestations of otherwise clinically less apparent cervical anastomotic leakages. This hypothesis is supported by the fact that following a transthoracic esophagectomy with cervical anastomosis, intrathoracic manifestations are more often observed compared to a transhiatal esophagectomy [20].

This study derives strength from its nationwide study design, which allowed for propensity score matching and analyses of a large number of esophageal cancer patients. Moreover, a benefit of this study is that the majority of the patients received neoadjuvant chemoradiation, which has been shown to improve long-term survival [5]. This aligns with current standard practice, making our findings relevant and generalizable to today’s patient population, thereby enhancing the practical value of our conclusions. However, several limitations should be noted. First, patients who underwent surgery in the year 2015 had to be excluded, as the extent of the mediastinal lymphadenectomy was not registered in the DUCA in that year. Second, it was not possible to account for the biological nature of the cancer, such as MSS/MSI status and PDL-1 expression, due to the lack of these data in the DUCA database. Furthermore, current evidence suggests that the lymph node metastasis areas of upper, middle and lower esophageal cancer are slightly different. Unfortunately, the exact location and number of lymph node metastasis were not available and therefore this could not be incorporated into the analyses. Also, data on cause of death was not available, which makes it impossible to provide cancer-specific survival information. Additionally, the lower percentage of N2/N3 stages in the adenocarcinoma group could contribute to the similar survival rates observed across different extents of lymphadenectomy. Moreover, the link between Vektis data and DUCA data was made in 2017; the follow-up period was therefore relatively short for the patients who underwent surgery in more recent years, and potentially this follow-up time has been too short to fully capture the long-term benefits of a paratracheal lymphadenectomy. In addition, in the DUCA database, postoperative complications are only registered within a 30-day postoperative period. Postoperative complications after this period are therefore not registered. Furthermore, a limitation of propensity score matching is that the choice of variables to match on influences study population size and study outcomes. For example, T- and N-stage were included as matching variables to avoid them acting as a confounder. While including both early and late-stage tumors provides sufficient study power, it is essential to consider that our results apply to a heterogeneous patient group. Moreover, propensity score matching could only be based on variables that were available in the DUCA database. As such, hospital-specific factors—that are not registered in the DUCA database—could not be included. This makes the study population more heterogeneous and might have contributed to residual bias, as most hospitals perform a paratracheal lymphadenectomy on indication, while it is standard procedure in a few other hospitals. This means that most probably the technical skills to perform a proper paratracheal lymphadenectomy differ between Dutch hospitals, which may in part explain the increased occurrence of some complications following paratracheal lymphadenectomy. Furthermore, this may also explain the difference in oncological outcomes in terms of survival compared to results from the studies from the East.

## 5. Conclusions

In conclusion, this nationwide study demonstrated that the addition of paratracheal lymphadenectomy significantly increases lymph node yield in transthoracic esophagectomy, which did not translate into significantly improved survival for esophageal cancer patients in the current dataset. However, a higher rate of surgical morbidity was seen in these patients. Whether these results remain when paratracheal lymphadenectomy is performed as standard procedure in all esophageal cancer patients requires further investigation.

## Figures and Tables

**Table 1:** Patient and treatment characteristics of patients with adenocarcinoma, both before and after propensity score matching.

	Before Propensity Score Matching					After Propensity Score Matching				
	Paratrachael Lymphadenectomy					Paratrachael Lymphadenectomy				
	Yes (*n* = 959)		No (*n* = 748)			Yes (*n* = 577)		No (*n* = 577)		
	N	(%)	N	(%)	SMD	N	(%)	N	(%)	SMD
Gender, female	152	(16)	124	(17)	0.020	95	(17)	94	(16)	0.005
Age, mean (SD)	64	(10)	64	(9)	0.088	64	(10)	65	(9)	0.090
BMI, mean (SD)	26	(4)	27	(4)	0.078	27	(4)	27	(4)	0.074
Comorbidity										
Pulmonary	149	(16)	125	(17)	0.032	96	(17)	88	(15)	0.038
Cardiac	201	(21)	162	(22)	0.017	118	(21)	134	(23)	0.067
Vascular	348	(36)	247	(33)	0.069	193	(33)	190	(33)	0.011
Diabetes	148	(15)	118	(16)	0.009	92	(16)	96	(17)	0.019
Neurological	124	(13)	80	(11)	0.069	70	(12)	58	(10)	0.066
Previous thoracic or abdominal surgery	296	(31)	206	(28)	0.073	171	(30)	158	(27)	0.050
ASA score					0.145					0.098
I	166	(17)	131	(18)		106	(18)	102	(18)	
II	584	(61)	491	(66)		356	(62)	380	(66)	
III	206	(22)	126	(16)		115	(20)	95	(16)	
IV	3	(<1)	0			0		0		
Tumor location					0.100					0.045
Upper or Middle third	67	(7)	37	(5)		31	(5)	36	(6)	
Distal third	698	(73)	541	(72)		420	(73)	410	(71)	
GEJ	194	(20)	170	(23)		126	(22)	131	(23)	
Year of surgery					0.420					0.039
2011–2012	318	(33)	122	(16)		104	(18)	108	(19)	
2013–2014	349	(36)	294	(39)		239	(41)	228	(39)	
2016–2017	292	(31)	332	(45)		234	(41)	241	(42)	
Location of anastomosis					0.762					0.070
Cervical	576	(61)	186	(25)		202	(35)	183	(32)	
Intrathoracic	383	(39)	562	(75)		375	(65)	394	(68)	
Clinical T stage					0.054					0.067
cT1	34	(4)	20	(3)		21	(4)	16	(3)	
cT2	173	(18)	131	(17)		107	(18)	98	(17)	
cT3	686	(71)	546	(73)		411	(71)	424	(73)	
cT4	66	(7)	51	(7)		38	(7)	39	(7)	
Clinical N stage					0.153					0.067
cN0	313	(33)	275	(37)		211	(37)	202	(35)	
cN1	398	(42)	317	(42)		230	(40)	244	(42)	
cN2	209	(21)	141	(19)		123	(21)	118	(21)	
cN3	39	(4)	15	(2)		13	(2)	13	(2)	
Neoadjuvant treatment	902	(94)	696	(93)	0.041	534	(93)	535	(93)	0.007

BMI; Body Mass Index. ASA; American Society of Anesthesiologists. GEJ; gastroesophageal junction. SMD; standardized mean difference. Clinical T and N staging was performed according to the American Joint Committee on Cancer (AJCC) staging system.

**Table 2:** Clinical and pathological results of patients with adenocarcinoma with paratracheal lymphadenectomy versus without paratracheal lymphadenectomy after propensity score matching.

	Paratrachael Lymphadenectomy				
	Yes (*n* = 577)		No (*n* = 577)		
	N	(%)	N	(%)	*p*-Value
Lymph node yield, median [IQR]					
Total	22	(17–30)	19	(15–25)	<0.001
Tumor-positive	0	(0–2)	0	(0–2)	0.169
pN stage					0.373
N0	336	(58)	363	(63)	
N1	125	(22)	105	(18)	
N2	74	(13)	67	(12)	
N3	42	(7)	42	(7)	
Unknown					
Radicality					0.395
R0	549	(95)	539	(94)	
R1–2	26	(5)	37	(6)	
Unknown	2	(<1)	1	(<1)	
Postoperative complications					
Yes, any	350	(61)	347	(60)	0.857
Recurrent laryngeal nerve injury	59	(10)	30	(5)	0.002
Anastomotic leakage	146	(25)	145	(25)	0.946
Pulmonary complications	185	(32)	199	(35)	0.417
Chylothorax	55	(10)	31	(5)	0.010
Re-interventions					
Yes, any	152	(26)	142	(25)	0.543
Radiological	58	(10)	61	(11)	0.847
Endoscopic	67	(12)	54	(9)	0.933
Re-operation	81	(14)	82	(14)	0.933
Length of hospital stay, median [IQR]	13	(10–22)	13	(9–19)	0.515
Mortality (in-hospital or within 30 days after surgery)	12	(2)	20	(4)	0.289

**Table 3:** Patient and treatment characteristics of patients with squamous cell carcinoma, both before and after propensity score matching.

	Before Propensity Score Matching					After Propensity Score Matching				
	Paratrachael Lymphadenectomy					Paratrachael Lymphadenectomy				
	Yes (*n* = 363)		No (*n* = 165)			Yes (*n* = 147)		No (*n* = 147)		
	N	(%)	N	(%)	SMD	N	(%)	N	(%)	SMD
Gender, female	189	(52)	78	(47)	0.096	78	(53)	71	(48)	0.095
Age, mean (SD)	64	(9)	64	(10)	0.001	64	(9)	61	(10)	0.025
BMI, mean (SD)	23	(4)	23	(4)	0.020	23	(4)	23	(4)	0.035
Previous thoracic or abdominal surgery	119	(33)	54	(33)	0.001	46	(31)	44	(30)	0.030
ASA score					0.099					0.069
I	58	(16)	30	(18)		30	(20)	29	(20)	
II	221	(61)	103	(63)		91	(62)	88	(60)	
III	84	(23)	32	(19)		26	(18)	30	(20)	
Tumor location					0.335					0.054
Upper or Middle third	229	(73)	77	(47)		70	(48)	74	(50)	
Distal third—GEJ	134	(37)	88	(53)		77	(52)	73	(50)	
Year of surgery					0.434					0.066
2011–2012	130	(36)	37	(22)		39	(26)	35	(24)	
2013–2014	139	(38)	53	(32)		48	(33)	51	(34)	
2016–2017	94	(26)	75	(46)		60	(41)	61	(42)	
Location of anastomosis					0.648					0.042
Cervical	305	(84)	92	(56)		95	(65)	92	(63)	
Intrathoracic	58	(16)	73	(44)		52	(35)	55	(37)	
Clinical T stage					0.111					0.069
cT1–2	87	(24)	32	(19)		26	(17)	30	(20)	
cT3–4	276	(76)	133	(81)		121	(83)	117	(80)	
Clinical N stage					0.015					0.059
cN0	114	(31)	53	(32)		44	(30)	48	(33)	
cN+	249	(69)	112	(68)		103	(70)	99	(67)	
Neoadjuvant treatment	339	(93)	154	(93)	0.002	137	(93)	136	(93)	0.026

BMI; Body Mass Index. ASA; American Society of Anesthesiologists. GEJ; gastroesophageal junction. SMD; standardized mean difference. Clinical T and N staging was performed according to the American Joint Committee on Cancer (AJCC) staging system.

**Table 4:** Clinical and pathological results of patients with squamous cell carcinoma with paratracheal lymphadenectomy versus without paratracheal lymphadenectomy after propensity score matching.

	Paratrachael Lymphadenectomy				
	Yes (*n* = 147)		No (*n* = 147)		
	N	(%)	N	(%)	*p*-Value
Lymph node yield, median [IQR]					
Total	22	(16–30)	19	(13–26)	0.010
Tumor-positive	0	(0–1)	0	(0–1)	0.867
pN stage					0.965
N0	103	(70)	101	(69)	
N1	32	(22)	34	(23)	
N2	8	(5)	9	(6)	
N3	4	(3)	3	(2)	
Radicality					0.574
R0	142	(97)	142	(97)	
R1–2	5	(3)	4	(3)	
Unknown	0		1	(<1)	
Postoperative complications					
Yes, any	97	(66)	90	(61)	0.396
Recurrent laryngeal nerve injury	8	(5)	11	(8)	0.477
Anastomotic leakage	62	(42)	41	(27)	0.014
Pulmonary complications	51	(35)	50	(34)	0.902
Chylothorax	19	(13)	13	(9)	0.261
Re-interventions					
Yes, any	42	(29)	28	(19)	0.075
Radiological	16	(11)	11	(8)	0.420
Endoscopic	15	(10)	9	(6)	0.287
Re-operation	20	(14)	13	(9)	0.267
Length of hospital stay, median [IQR]	14	(10–26)	12	(9–17)	0.004
Mortality (in-hospital or within 30 days after surgery)	7	(5)	4	(3)	0.541

## Data Availability

Restrictions apply to the availability of these data. Data were obtained from Dutch Upper GI Cancer Audit (DUCA) and VEKTIS and are available at https://dica.nl/data-aanvragen/ (date accessed 1 January 2019) with the permission of the DUCA scientific committee.

## References

[1] Lordick F., Mariette C., Haustermans K., Obermannová R., Arnold D. (2016). Oesophageal cancer: ESMO Clinical Practice Guidelines for diagnosis, treatment and follow-up. Ann. Oncol..

[2] Obermannová R., Alsina M., Cervantes A., Leong T., Lordick F., Nilsson M., van Grieken N., Vogel A., Smyth E.C., ESMO Guidelines Committee (2022). Oesophageal cancer: ESMO Clinical Practice Guideline for diagnosis, treatment and follow-up. Ann. Oncol..

[3] Ng C.B., Chiu C.-H., Yeh C.-J., Chang Y.-C., Hou M.-M., Tseng C.-K., Liu Y.-H., Chao Y.-K. (2024). Temporal Trends in Survival Outcomes for Patients with Esophageal Cancer Following Neoadjuvant Chemoradiotherapy: A 14-Year Analysis. Ann. Surg. Oncol..

[4] Biere S.S., van Berge Henegouwen M.I., Maas K.W., Bonavina L., Rosman C., Garcia J.R., Gisbertz S.S., Klinkenbijl J.H., Hollmann M.W., de Lange E.S. (2012). Minimally invasive versus open oesophagectomy for patients with oesophageal cancer: A multicentre, open-label, randomised controlled trial. Lancet.

[5] Shapiro J., van Lanschot J.J.B., Hulshof M.C.C.M., van Hagen P., van Berge Henegouwen M.I., Wijnhoven B.P.L., van Laarhoven H.W.M., Nieuwenhuijzen G.A.P., Hospers G.A.P., Bonenkamp J.J. (2015). Neoadjuvant chemoradiotherapy plus surgery versus surgery alone for oesophageal or junctional cancer (CROSS): Long-term results of a randomised controlled trial. Lancet Oncol..

[6] Low D.E., Kuppusamy M.K., Alderson D., Cecconello I., Chang A.C., Darling G., Davies A., D’journo X.B., Gisbertz S.S., Griffin S.M. (2019). Benchmarking Complications Associated with Esophagectomy. Ann. Surg..

[7] Xia L., Shi W., Cai Y., Liao Z.M., Huang Z., Qiu H., Wang J., Chen Y. (2024). Comparison of long-term survival of neoadjuvant therapy plus surgery versus upfront surgery and the role of adjuvant therapy for T1b-2N0-1 esophageal cancer: A population study of the SEER database and Chinese cohort. Int. J. Surg..

[8] Gupta V., Coburn N., Kidane B., Hess K.R., Compton C., Ringash J., Darling G., Mahar A.L. (2018). Survival prediction tools for esophageal and gastroesophageal junction cancer: A systematic review. J. Thorac. Cardiovasc. Surg..

[9] Yamasaki M., Miyata H., Miyazaki Y., Takahashi T., Kurokawa Y., Nakajima K., Takiguchi S., Mori M., Doki Y. (2014). Evaluation of the nodal status in the 7th edition of the UICC-TNM classification for esophageal squamous cell carcinoma: Proposed modifications for improved survival stratification: Impact of lymph node metastases on overall survival after esophagectomy. Ann. Surg. Oncol..

[10] Miyata H., Sugimura K., Yamasaki M., Makino T., Tanaka K., Morii E., Omori T., Yamamoto K., Yanagimoto Y., Yano M. (2018). Clinical Impact of the Location of Lymph Node Metastases After Neoadjuvant Chemotherapy for Middle and Lower Thoracic Esophageal Cancer. Ann. Surg. Oncol..

[11] Phillips A.W., Lagarde S.M., Navidi M., Disep B., Griffin S.M. (2017). Impact of Extent of Lymphadenectomy on Survival, Post Neoadjuvant Chemotherapy and Transthoracic Esophagectomy. Ann. Surg..

[12] DICA Jaarrapportage 2018 [Internet]. https://dica.nl/.

[13] Park S.Y., Kim D.J., Son T., Lee Y.C., Lee C.Y., Lee J.G., Chung K.Y. (2017). Extent of Mediastinal Lymphadenectomy and Survival in Superficial Esophageal Squamous Cell Carcinoma. J. Gastrointest. Surg..

[14] Kingma B., Hagens E., van Berge Henegouwen M., Borggreve A., Ruurde J., Gisbertz S., van Hillegersberg R. (2023). The impact of paratracheal lymphadenectomy on nodal yield and clinical outcomes in esophagectomy for cancer: A nation-wide propensity score-matched analysis. Dig. Surg..

[15] Vectis Vektis [Internet]. https://www.vektis.nl/.

[16] Lonjon G., Porcher R., Ergina P., Fouet M., Boutron I. (2017). Potential Pitfalls of Reporting and Bias in Observational Studies with Propensity Score Analysis Assessing a Surgical Procedure: A Methodological Systematic Review. Ann. Surg..

[17] Visser E., Markar S.R., Ruurda J.P., Hanna G.B., van Hillegersberg R. (2019). Prognostic Value of Lymph Node Yield on Overall Survival in Esophageal Cancer Patients: A Systematic Review and Meta-analysis. Ann Surg..

[18] Voeten D.M., Busweiler L.A.D., van der Werf L.R., Wijnhoven B.P.L., Verhoeven R.H.A., van Sandick J.W., van Hillegersberg R., van Berge Henegouwen M.I., Dutch Upper Gastrointestinal Cancer Audit (DUCA) Group (2021). Outcomes of Esophagogastric Cancer Surgery During Eight Years of Surgical Auditing by the Dutch Upper Gastrointestinal Cancer Audit (DUCA). Ann Surg..

[19] Visser E., van Rossum P.S.N., Ruurda J.P., van Hillegersberg R. (2017). Impact of Lymph Node Yield on Overall Survival in Patients Treated with Neoadjuvant Chemoradiotherapy Followed by Esophagectomy for Cancer. Ann. Surg..

[20] Tachimori Y., Ozawa S., Numasaki H., Matsubara H., Shinoda M., Toh Y., Udagawa H., Fujishiro M., Oyama T., The Registration Committee for Esophageal Cancer of the Japan Esophageal Society (2015). Efficacy of lymph node dissection by node zones according to tumor location for esophageal squamous cell carcinoma. Esophagus.

[21] Kosugi S.-I., Ichikawa H., Hanyu T., Ishikawa T., Wakai T. (2017). Appropriate extent of lymphadenectomy for squamous cell carcinoma of the esophagogastric junction. Int. J. Surg..

[22] Harada K., Hwang H., Wang X., Abdelhakeem A., Iwatsuki M., Murphy M.A.B., Maru D.M., Weston B., Lee J.H., Rogers J.E. (2019). Frequency and Implications of Paratracheal Lymph Node Metastases in Resectable Esophageal or Gastroesophageal Junction Adenocarcinoma. Ann. Surg..

[23] Kurokawa Y., Hiki N., Yoshikawa T., Kishi K., Ito Y., Ohi M., Wada N., Takiguchi S., Mine S., Hasegawa S. (2015). Mediastinal lymph node metastasis and recurrence in adenocarcinoma of the esophagogastric junction. Surgery.

[24] KKurokawa Y., Takeuchi H., Doki Y., Mine S., Terashima M., Yasuda T., Yoshida K., Daiko H., Sakuramoto S., Yoshikawa T. (2021). Mapping of Lymph Node Metastasis from Esophagogastric Junction Tumors: A Prospective Nationwide Multicenter Study. Ann. Surg..

[25] Taniyama Y., Miyata G., Kamei T., Nakano T., Abe S., Katsura K., Sakurai T., Teshima J., Hikage M., Ohuchi N. (2014). Complications following recurrent laryngeal nerve lymph node dissection in oesophageal cancer surgery. Interact. Cardiovasc. Thorac. Surg..

[26] Tan Z., Ma G., Zhao J., Bella A.E., Rong T., Fu J., Meng Y., Luo K., Situ D., Lin P. (2014). Impact of Thoracic Recurrent Laryngeal Node Dissection: 508 Patients with Tri-Incisional Esophagectomy. J. Gastrointest. Surg..

[27] van Rijswijk A.S., Hagens E.R.C., van der Peet D.L., Henegouwen M.I.V.B., Gisbertz S.S. (2019). Differences in Esophageal Cancer Surgery in Terms of Surgical Approach and Extent of Lymphadenectomy: Findings of an International Survey. Ann. Surg. Oncol..

[28] Ketel M.H.M., van der Aa D.C., Henckens S.P.G., Rosman C., Henegouwen M.I.v.B., Klarenbeek B.R., Gisbertz S.S., DES Collaboration Group (2024). Extent and Boundaries of Lymph Node Stations During Minimally Invasive Esophagectomy: A Survey Among Dutch Esophageal Surgeons. Ann. Surg. Oncol..

[29] Van Heijl M., Van Wijngaarden A.K.S., Lagarde S.M., Busch O.R.C., Van Lanschot J.J.B., Van Berge Henegouwen M.I. (2010). Intrathoracic manifestations of cervical anastomotic leaks after transhiatal and transthoracic oesophagectomy. Br. J. Surg..

